# Differences in initial electrocardiographic findings between ST-elevation myocardial infarction due to left main trunk and left anterior descending artery lesions

**DOI:** 10.1186/s12245-019-0227-x

**Published:** 2019-04-05

**Authors:** Toshiharu Fujii, Misaki Hasegawa, Junichi Miyamoto, Yuji Ikari

**Affiliations:** Department of Cardiology, Division of Cardiovascular Medicine, Tokai University School of Medicine, 143 Shimokasuya, Isehara, 259-1193 Japan

**Keywords:** Electrocardiography, ST-elevation myocardial infarction, Left main trunk, Left anterior descending artery, ST-segment elevation, ST-segment depression

## Abstract

**Background:**

Early discrimination of ST-elevation myocardial infarction (STEMI) due to a left main trunk (LMT) lesion provided by straightforward electrocardiographic criteria is useful for prompt treatment. The purpose of this study is to investigate differences in electrocardiographic findings between STEMI due to lesions of LMT and those of left anterior descending artery (LAD).

**Methods:**

Initial electrocardiogram (ECG) recordings of 435 patients with analyzable ECGs from a cohort of 940 consecutive STEMI patients were analyzed retrospectively for presence of LMT lesions (LMT, *n* = 39), proximal (pLAD, *n* = 224) and distal LAD lesions (dLAD, *n* = 172). ST-segment deviations in 12 leads were assessed among 3 groups without bundle branch block (*n* = 17 in LMT, *n* = 180 in pLAD, and *n* = 159 in dLAD).

**Results:**

Magnitudes of ST-segment deviations showed significant differences in leads II, III, aVR aVL, aVF, and V2–V6 across the three groups. This difference suggested two possible characteristic findings in the LMT group, allowing it to be distinguished from the pLAD or dLAD group; (A) larger magnitude of ST-segment depression in lead II than that of ST-segment elevation in lead V2 (47.1% in LMT vs. 0.6% in pLAD vs. 1.3% in dLAD, *P* < 0.0001), and (B) ST-segment depression in lead V5 (58.8% in LMT vs. 6.7% in pLAD vs. 2.5% in dLAD, *P* < 0.0001). These findings exhibited superior negative predictive value over conventional ST-segment elevation in lead aVR.

**Conclusions:**

A large reciprocal ST-segment depression in inferior leads and ST-segment depression in lead V5 are useful ECG findings allowing determination of STEMI due to an LMT lesion.

## Background

Current prompt revascularization by primary percutaneous coronary intervention and optimized medical therapy for ST-elevation myocardial infarction (STEMI) have resulted in dramatic improvement of patient mortality [1]. This improvement provided by serial medical interventions, beginning with first medical contact, has increased in recent years. On the other hand, since short-term mortality in STEMI caused by left main trunk (LMT) lesions remains high, faster diagnosis and medical intervention is required to improve patient survival [2, 3].

Conventional electrocardiography remains the gold standard modality for early diagnosis of STEMI, even in the current advanced diagnostic modality era. The Electrocardiogram (ECG) plays an important role in diagnosis and identification of the location of the culprit infarct artery, and its diagnostic accuracy is reported to be high [4].

**Fig. 1:**
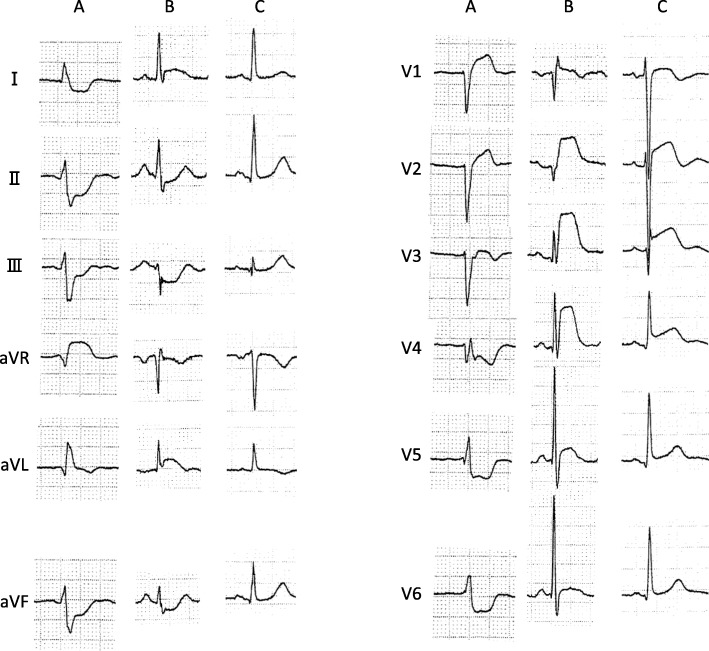
Representative electrocardiograms. **a** STEMI due to LMT lesion, **b** STEMI due to proximal LAD lesion, and **c** STEMI due to distal LAD lesion

However, it is not always easy to confirm that the culprit lesion is an LMT lesion, based only on initial ECG findings; it is especially difficult to distinguish an LMT lesion from a left anterior descending artery (LAD) lesion (Fig. 1). Definition of differences in respective ECG findings of these two lesions may help choose a more appropriate medical intervention strategy and thus improve short term-mortality [5].

To investigate electrocardiographic differences in the initial ECG that may distinguish STEMI due to an LMT lesion from those due to an LAD lesion, electrocardiographic parameters were compared among the following three groups; LMT, proximal LAD, and distal LAD lesions.

## Methods

### Study design and population

**Fig. 2:**
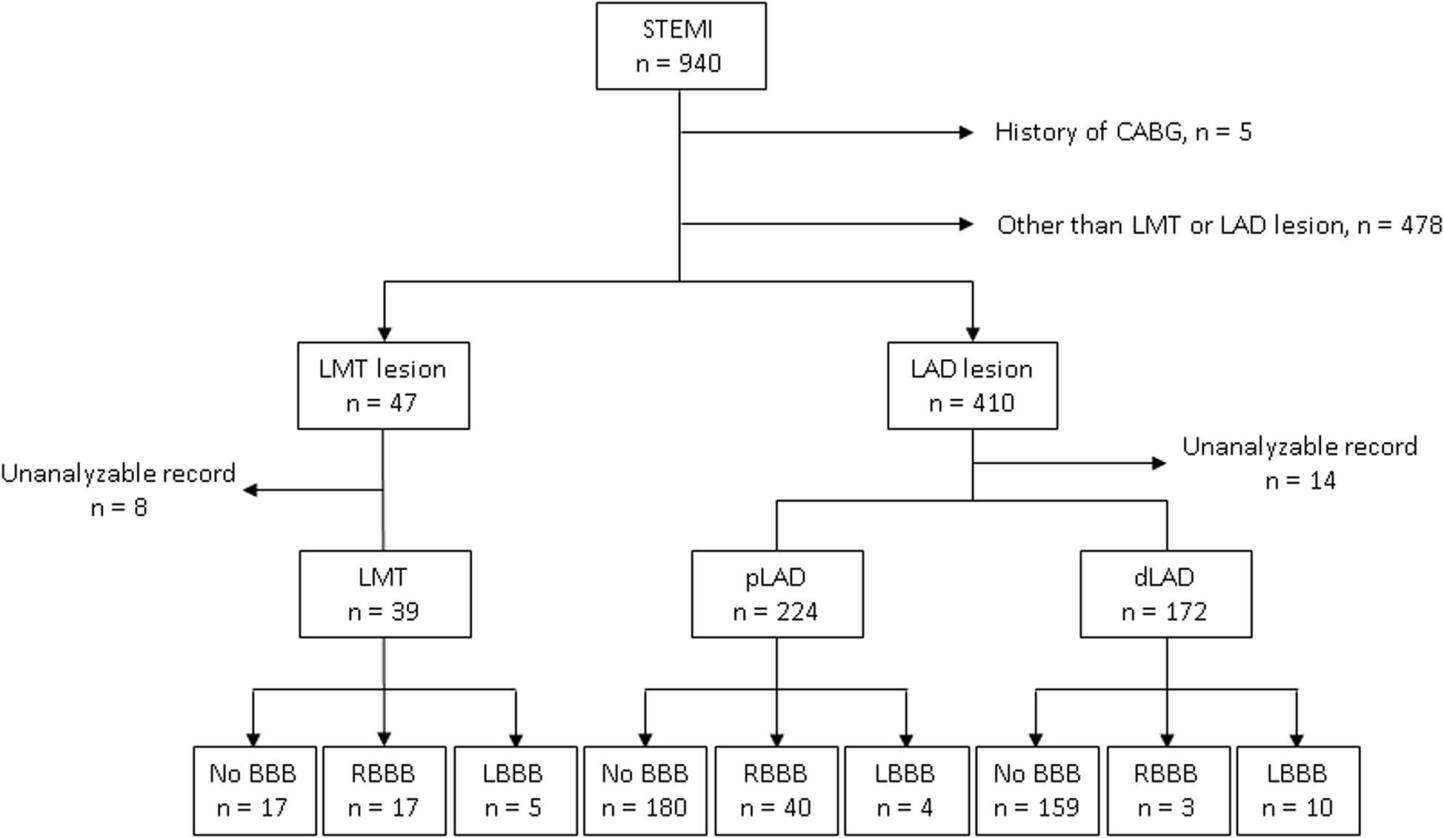
Flow diagram describing patient selection

To identify electrocardiographic differences that may distinguish STEMI due to an LMT lesion from that due to an LAD lesion, initial ECG parameters were compared between STEMI patients with LMT lesions and those with LAD lesions. The present study retrospectively surveyed 940 STEMI patients whose culprit lesions were confirmed by emergency coronary angiography within 24 h of symptom onset, from January 2006 to March 2017, at Tokai University School of Medicine. Five patients who had undergone coronary artery bypass grafting were excluded. There were 47 patients with STEMI due to LMT lesions and 410 patients with STEMI due to LAD lesion. Among these, 8 patients with LMT lesions and 14 patients with LAD lesions were excluded because of lost or unanalyzable ECGs. Thirty-nine patients in the LMT lesion group, 224 patients in the proximal LAD lesion (pLAD) group, and 172 patients in the distal LAD lesion (dLAD) were ultimately analyzed. Each group was divided into three sub-groups on the basis of presence or absence of right (RBBB) or left bundle branch block (LBBB): (1) normal QRS without bundle branch block (No BBB), (2) RBBB, and (3) LBBB. The flow diagram describing patient selection is summarized in Fig. 2.

The present study was approved by the institutional review board for Clinical Research of the General Clinical Research Center in Tokai University School of Medicine.

### Definitions

STEMI was defined according to the Third universal definition of myocardial infarction [6]. Infarction-related culprit artery and final diagnosis were judged by emergency coronary angiography performed within 24 h of symptom onset.

LAD lesions were divided into proximal and distal groups. The lesion proximal to the first septal branch was defined as proximal LAD, and the lesion distal to this branch was defined as distal LAD. To determine concomitant coronary artery disease, significant coronary artery disease was defined as more than 70% stenosis in a main epicardial coronary artery in at least one view by angiographic evaluation.

A standard 12-lead resting surface ECG was taken on hospital arrival (paper speed: 25 mm/s, calibration: 1 mV = 10 mm). This initial ECG which was recorded after hospital arrival and before emergency coronary angiography was required to meet the following ECG inclusion criteria: sinus rhythm and an analyzable ECG record. Left anterior hemiblock (LAHB) was defined as a qR pattern in aVL leads and left axis deviation less than − 45°. QT interval was measured from the beginning of the QRS complex to the end of the T wave using the lead with the longest duration. QT intervals were corrected for heart rate according to Bazett’s and Fridericia’s formula (QTc) [7]. ST-segment deviation was assessed at the J-point in patients without RBBB or LBBB. Baseline level of ST-segment was defined as 0 mV and was defined as positive in the upper direction (elevation) or negative in the lower direction (depression).

### Statistical analysis

Numerical factors with skewed distribution were shown as medians (interquartile range). The Kruskal-Wallis test was used to determine statistically significant differences in clinical parameters among the three groups. Steel tests were used for multiple comparisons of ST-segment deviation in the LMT group with that in the pLAD or dLAD group. Fisher’s exact test was used to determine differences in categorical variables. Positive predictive values (PPV) and negative predictive values (NPV) were used to assess the diagnostic accuracy of ECG criteria for evaluation of an LMT lesion. A *P* value < 0.05 was considered statistically significant. *P* values in the tables show the statistical comparison among the three groups. All statistical calculations were performed using JMP version 11 (SAS Institute, Inc.; Cary, NC, USA).

## Results

To identify electrocardiographic differences allowing distinction of STEMI due to an LMT lesion from that due to an LAD lesion, electrocardiographic parameters were assessed quantitatively. Baseline characteristics among the LMT, pLAD, and dLAD groups are presented in Table 1. Patients in the LMT group were more likely to have hypertension, low hemoglobin, low LDL-cholesterol, low triglyceride, and impaired renal function. Patients in whom hemodynamic instability developed at onset, vital shock and heart failure, constituted a higher proportion in the LMT group than the pLAD and dLAD groups.

**Table 1:** Baseline characteristics and clinical status on arrival

	LMT, *n* = 39	pLAD, *n* = 224	dLAD, *n* = 172	*P* value
Age, year	71 (62, 77)	66.5 (56, 75)	66 (59.3, 75)	0.1786
Male, *n*	29 (74.4%)	182 (81.3%)	136 (79.1%)	0.5875
Height, cm	163 (154.7, 169)	164.4 (158, 170)	164 (158, 168)	0.3621
Weight, kg	60 (55, 70)	65 (55, 72)	63 (53.8, 72)	0.3751
Current smoking, *n*	9 (23.1%)	80 (35.7%)	59 (34.3%)	0.4282
Hypertension, *n*	35 (89.7%)	158 (70.5%)	137 (79.7%)	0.0115
Dyslipidemia, *n*	23 (59.0%)	160 (71.4%)	128 (74.4%)	0.1555
Diabetes mellitus, *n*	16 (41.0%)	82 (36.6%)	61 (35.5%)	0.8088
Insulin therapy	2 (5.1%)	12 (5.4%)	9 (5.2%)	0.9974
Hemodialysis, *n*	1 (2.6%)	3 (1.3%)	3 (1.7%)	0.8405
Hemoglobin, mg/dl	13.7 (11.2, 15.3)	14.8 (13.4, 16.1)	14.4 (13, 15.9)	0.0134
LDL-chol, mg/d	108.5 (88.3, 133.5)	123 (101, 150)	129 (106.8, 155.3)	0.00171
HDL-chol, mg/dl	45.5 (40, 58.3)	49 (39, 57)	47 (39, 58)	0.9678
Triglyceride, mg/dl	84 (40.8, 110)	103 (59, 170)	81 (52.5, 141)	0.0169
Serum creatinine, mg/dl	1.2 (0.9, 1.5)	0.9 (0.7, 1.1)	0.8 (0.7, 1.0)	< 0.0001
eGFR, ml/min/1.73 m^2^	46.0 (35.0, 64.3)	64.7 (51.7, 79.9)	68.2 (53.7, 82.5)	< 0.0001
Shock on arrival, *n*	29 (74.4%)	36 (16.1%)	17 (9.9%)	< 0.0001
Killip I, *n*	1 (2.6%)	89 (39.7%)	94 (54.7%)	< 0.0001
Time of onset to ECG, min	60.5 (39.8, 230.6)	78 (45.8, 195.3)	82 (48.2, 220.0)	0.1369
Peak CPK, IU/l	11,249 (2961.3, 18,027)	4059 (2021, 6115)	2369 (1174.5, 4358.5)	< 0.0001
LVEF, %	35.5 (29.0, 45.0)	48.5 (41, 54.8)	51 (44, 58)	< 0.0001

*eGFR* estimated glomerular filtration rate, *ECG* electrocardiogram, *CPK* creatine phosphokinase, *LVEF* left ventricular ejection fraction

### Comparison of electrocardiographic parameters

Table 2 shows the comparison of electrocardiographic parameters across the three groups. The percentage of patients with RBBB and LBBB was 43.6% (17/39) and 12.8% (5/39) in the LMT group, 17.9% (40/224) and 1.8% (4/224) in the pLAD group, and 1.7% (3/172) and 5.8% (10/172) in the dLAD group respectively. QRS duration was significantly longer in the LMT group than in the pLAD and dLAD groups without bundle branch block (110 ms vs. 96 ms vs. 92 ms, *P* = 0.0031). QTc intervals, calculated from Bazett’s and Fridericia formula, were significantly longer in the LMT group than in the pLAD and dLAD groups without bundle branch block (Bazett, 481.2 ms vs. 446.0 ms vs. 449.9 ms, *P* = 0.0021, respectively; Fridericia, 450.1 ms vs. 425.3 ms vs. 431.9 ms, *P* = 0.0041, respectively).

**Table 2:** Assessment of electrocardiographic parameters

	LMT, *n* = 39			pLAD, *n* = 224			dLAD, *n* = 172			*P* value		
	Normal *n* = 17	RBBB *n* = 17	LBBB *n* = 5	Normal *n* = 180	RBBB *n* = 40	LBBB *n* = 4	Normal *n* = 159	RBBB *n* = 3	LBBB *n* = 10	Normal	RBBB	LBBB
Heart rate, bpm	93 (79, 128)	86 (72, 97.5)	76 (57.5, 119.5)	78 (65, 90.8)	87 (73.3, 113)	130.5 (84.8, 147.8)	77 (66, 93)	73 (69, 96)	81 (66.3, 97)	0.0092	0.5230	0.1628
QRS duration, ms	110 (95, 116)	156 (141.5, 162)	174 (160, 175)	96 (86.5, 106)	149 (131, 158)	162 (140, 208)	92 (87, 102)	134 (128, 146)	142 (135, 177.5)	0.0031	0.1933	0.2797
QRS axis	13 (−46.5, 60)	46 (−69, 63.5)	−16 (−48, 32)	43.5 (6.9, 68)	19.5 (−57.8, 70.8)	−54 (−78.5, 5.8)	33 (−9, 61)	- 47 (−52, 73)	−56.5 (−66, −44.5)	0.0369	0.6900	0.1473
LAHB, *n*	4 (23.5%)	9 (52.9%)	NA	10 (5.6%)	17 (42.5%)	NA	9 (5.7%)	2 (66.7%)	NA	0.0135	0.5975	NA
QT interval, ms	386 (366, 407)	428 (411, 446)	466 (417, 472)	395 (368, 415.5)	414 (382.5, 450.5)	403 (386.5, 484)	398 (368, 420)	438 (364, 458)	428 (408, 482.5)	0.7386	0.6695	0.6059
QTc, ms												
Bazett	481.2 (443.5, 560.1)	507.8 (482.9, 531.3)	524.6 (459.9, 587.7)	446.0 (424.7, 464.1)	503.0 (460.7, 541.1)	579.7 (565.8, 620.7)	449.9 (429.9, 477.7)	483.1 (460.4, 491.0)	507.8 (482.7, 565.8)	0.0021	0.5043	0.1755
Fridericia	450.1 (437.7, 496.3)	477.1 (457.1, 498.1)	504.3 (463.2, 524.2)	425.3 (410.5, 445.1)	473.0 (445.9, 499.8)	527.2 (508.6, 543.7)	431.9 (416.0, 448.4)	467.6 (425.7, 479.8)	477.9 (441.6, 535.6)	0.0041	0.6980	0.1600

*RBBB* right bundle branch block, *LBBB* left bundle branch block, *LAHB* left anterior hemiblock, *QTc* corrected QT

### ST-segment deviation in patients without bundle branch block

**Fig. 3:**
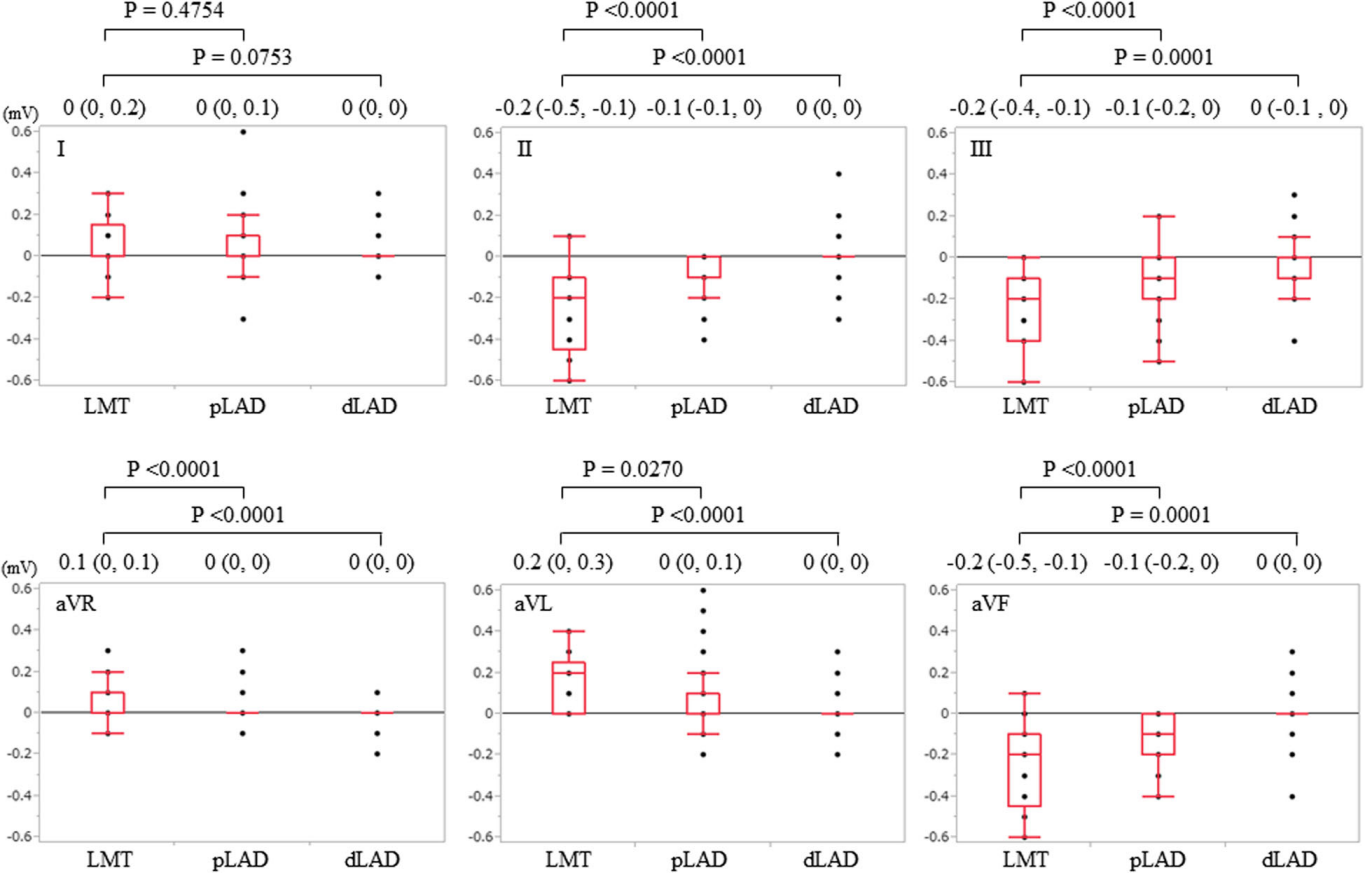
ST-segment deviation in limb leads. Quantitative ST-segment deviation in limb leads (I, II, III, aVR, aVL, aVF) is shown for the LMT, pLAD, and dLAD groups. ST-segment depression is shown in inferior leads (II, III, and aVF) in LMT and pLAD groups indicated as a reciprocal change of ST-segment elevation in precordial leads. The magnitude of ST-segment depression in the inferior leads is significantly greater in the LMT group than in the pLAD group. The dLAD group has less ST-segment depression in inferior leads

**Fig. 4:**
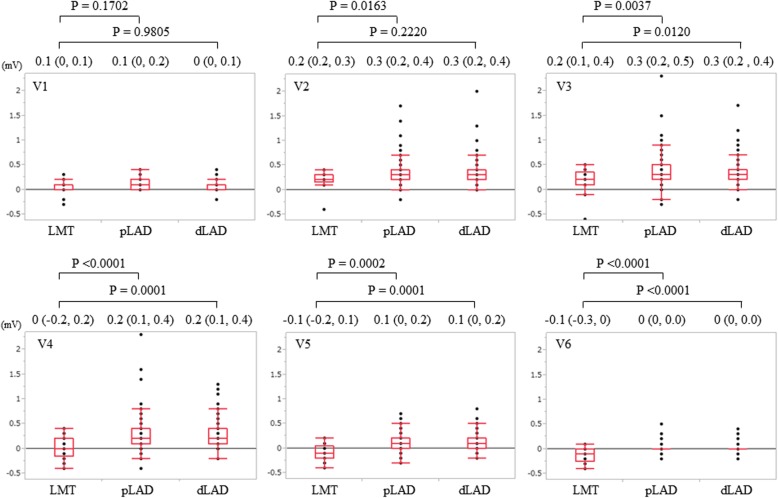
ST-segment deviation in precordial leads. Quantitative ST-segment deviation in precordial leads (V1–6) is shown for the LMT, pLAD, and dLAD groups. Elevated ST-segment is shown in leads V1–3 in the LMT group and in leads V1–5 in the pLAD and dLAD groups. The magnitude of ST-segment elevation in leads V2–V5 is significantly higher in the pLAD group than in the LMT group. ST-segment elevation in leads V2–3 is notable among the precordial leads in all three groups. While ST-level gradually decreased to 0 mV for lead V6 in the pLAD and dLAD groups, it became negative in leads V5–6 in the LMT group

Quantitative assessment of ST-segment deviation between LMT, pLAD, and dLAD groups in patients without RBBB or LBBB (17 patients in LMT group vs. 180 patients in pLAD group vs. 159 patients in dLAD group) is shown in Fig. 3 (limb leads; I, II, III, aVR aVL, aVF) and Fig. 4 (precordial leads; V1 to V6).

Among limb leads, ST-segment depression was shown in inferior leads (II, III, aVF) in LMT and pLAD groups as reciprocal change of ST-segment elevation in precordial leads (Fig. 3). The magnitude of ST-segment depression in the inferior leads was significantly greater in the LMT group than in the pLAD group and was significantly smaller in the dLAD group than that in the other two groups.

For precordial leads, the magnitude of ST-segment elevation in leads V2 to V5 was significantly lower in the LMT group than in the pLAD group, and that in leads V3 to V6 was significantly lower in the LMT group than in the dLAD group (Fig. 4). While ST-segment height gradually decreased to 0 mV for lead V6 in the pLAD and dLAD groups, it became negative in leads V5–6 in the LMT group. ST-segment in leads V5–6 was depressed in the LMT group as opposed to the pLAD and dLAD groups. ST-segment level in lead V5 showed a significant difference among the three groups [− 0.1 (− 0.2, 0.1) mV in LMT, 0.1 (0, 0.2) mV in pLAD, 0.1 (0, 0.2) mV in dLAD; *P* < 0.0001 for pLAD, *P* = 0.0001 for dLAD, respectively]. Furthermore, this level showed depression in the LMT group but elevation in the LAD group.

### Distinguishing criteria

#### ST-segment elevation in lead aVR

**Fig. 5:**
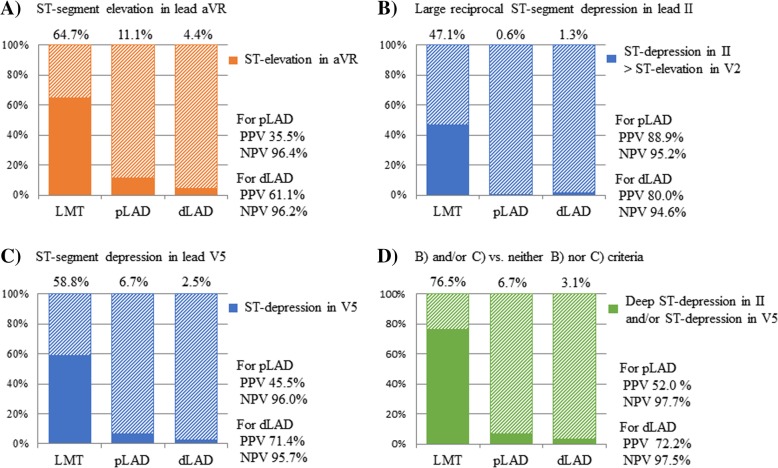
Diagnostic accuracy of novel suggested ECG criteria. The proportions of patients meeting each criterion with respect to ECG findings are shown.**a** ST-segment elevation in lead aVR. **b** Large reciprocal ST-segment depression in lead II. **c** ST-segment depression in lead V5. **d** (**b**) and/or (**c**) criteria vs. neither (**b**) nor (**c**) criteria: the proportion of patients who met criteria (**b**) and/or (**c**) vs. patients who met neither (**b**) nor (**c**) criteria are shown

ST-segment elevation in lead aVR is a well-known ECG finding in the diagnosis of an LMT lesion. We used this ECG criterion as a reference to evaluate the diagnostic accuracy of our proposed ECG criteria from (b) and (c) shown in Fig. 5. ST-segment elevation in lead aVR was observed in 64.7% (11/17) of patients in the LMT group, 11.1% (20/180) in the pLAD group, and 4.4% (7/159) in the dLAD group (*P* < 0.0001; Fig. 5a). PPV and NPV used to distinguish the LMT group from the pLAD and dLAD groups were 35.5% and 96.4%, 61.1% and 96.2%, respectively.

#### Large reciprocal ST-segment depression in lead II

Lead II had the largest magnitude of ST-segment depression among inferior leads, and lead V2 had the largest magnitude of ST-segment elevation among precordial leads. These leads were used for defining this ECG criterion. Comparing the absolute magnitude of ST-segment deviation between lead II and lead V2, the LMT group was found to have a high proportion of patients with larger magnitude of ST-segment deviation in lead II than in lead V2, namely “large reciprocal ST-segment depression in lead II” (47.1% in the LMT group vs. 0.6% in the pLAD group vs. 1.3% in the dLAD group, *P* < 0.0001, Fig. 5b). PPV and NPV, used to distinguish the LMT group from the pLAD and dLAD groups, were 88.9% and 95.2%, 80.0% and 94.6%, respectively.

#### ST-segment depression in lead V5

The LMT group had a significantly higher proportion of patients with ST-segment depression in lead V5 (58.5% in LMT group vs. 6.7% in pLAD group vs. 2.5% in the dLAD group, *P* < 0.0001; Fig. 5c). PPV and NPV, used to distinguish the LMT group from the pLAD and dLAD groups, were 45.5% and 96.0%, 71.4% and 95.7%, respectively.

#### (b) and/or (c) criteria vs. neither (b) nor (c) criteria

LMT, pLAD, and dLAD group were divided into the two groups according to above criteria (b) and (c); positive in (b) and/or (c) criteria vs. neither positive (b) nor (c) criteria. The LMT group had a significantly higher proportion of patients who satisfy (b) and/or (c) criteria than pLAD or dLAD group (76.5% in LMT group vs. 6.7% in pLAD group vs. 3.1% in the dLAD group, *P* < 0.0001; Fig. 5d). PPV and NPV, used to distinguish the LMT group from the pLAD and dLAD groups, were 52.0% and 97.7%, 72.2% and 97.5%, respectively.

### Distinguishing criteria in patients without concomitant coronary diseases

**Fig. 6:**
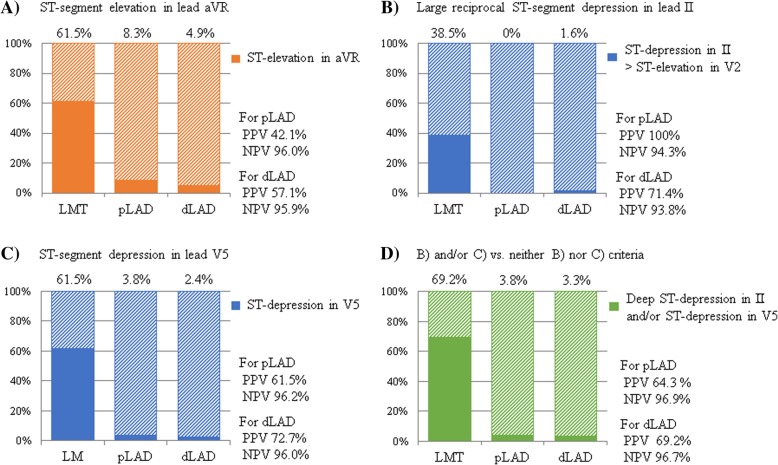
Diagnostic accuracy of novel suggested ECG criteria in patients without concomitant coronary artery diseases. **a** ST-segment elevation in lead aVR. **b** Large reciprocal ST-segment depression in lead II. **c** ST-segment depression in lead V5. **d** (**b**) and/or (**c**) criteria vs. neither (**b**) nor (**c**) criteria

To confirm diagnostic accuracy of our proposed ECG criteria in the patients without concomitant coronary artery disease other than culprit artery, the patients without concomitant coronary artery disease were extracted from each of LMT, pLAD, and dLAD group; patients without RCA lesion in LMT group (*n* = 13), patients without RCA, or LCX lesion in pLAD (*n* = 132) and dLAD group (*n* = 123) (Table 3). As well as Fig. 5, the proportion of these patients who satisfy the diagnostic criteria were demonstrated in Fig. 6.

**Table 3:** Concomitant significant coronary artery diseases

	1VD	2VD^a^		3VD
LMT				
No BBB, *n* = 17	0	13		4
RBBB, *n* = 17	0	16		1
LBBB, *n* = 5	0	4		1
pLAD				
No BBB, *n* = 180	132	RCA 18	LCX 20	10
RBBB, *n* = 40	26	RCA 2	LCX 5	7
LBBB, *n* = 4	2	RCA 0	LCX 0	2
dLAD				
No BBB, *n* = 159	123	RCA 9	LCX 15	12
RBBB, *n* = 3	2	RCA 0	LCX 1	0
LBBB, *n* = 10	4	RCA 3	LCX 2	2

*VD* vessel disease, *BBB* bundle branch block, *RBBB* right bundle branch block, *LBBB* left bundle branch block, *RCA* right coronary artery, *LCX* left circumflex artery

^a^LMT lesion was regarded as two vessel disease, LAD and LCX lesion

#### ST-segment elevation in lead aVR

ST-segment elevation in lead aVR was observed in 61.5% (8/13) of patients in the LMT group, 8.3% (11/132) in the pLAD group, and 4.9% (6/123) in the dLAD group (*P* < 0.0001; Fig. 6a). PPV and NPV, used to distinguish the LMT group from the pLAD and dLAD groups, were 42.1% and 96.0%, 57.1% and 95.9%, respectively.

#### Large reciprocal ST-segment depression in lead II

Large reciprocal ST-segment depression in lead II was observed in 38.5% (5/13) of patients in the LMT group, 0% (0/132) in the pLAD group, and 1.6% (2/123) in the dLAD group (*P* < 0.0001; Fig. 6b). PPV and NPV, used to distinguish the LMT group from the pLAD and dLAD groups, were 100% and 94.3%, 71.4% and 93.8%, respectively.

#### ST-segment depression in lead V5

ST-segment depression in lead V5 was observed in 61.5% (8/13) of patients in the LMT group, 3.8% (5/132) in the pLAD group, and 2.4% (3/123) in the dLAD group (*P* < 0.0001; Fig. 6c). PPV and NPV, used to distinguish the LMT group from the pLAD and dLAD groups, were 61.5% and 96.2%, 72.7% and 96.0%, respectively.

#### (b) and/or (c) criteria vs. neither (b) nor (c) criteria

The patients who met (b) and/or (c) criteria were observed in 69.2% (9/13) in the LMT group, 3.8% (5/132) in the pLAD group, and 3.3% (4/123) in the dLAD group (*P* < 0.0001; Fig. 6d). PPV and NPV, used to distinguish the LMT group from the pLAD and dLAD groups, were 64.3% and 96.9%, 69.2% and 96.7%, respectively.

## Discussion

The present study investigated electrocardiographic differences in STEMI due to LMT lesions versus STEMI due to LAD lesions, with a view to identification of distinguishing findings between such ECGs. A deep ST-segment depression in the inferior leads with low ST-segment elevation in the precordial leads, and ST-segment depression in V5–6 were distinctive findings in the LMT group as opposed to the proximal and distal LAD groups. A larger magnitude of ST-segment depression in lead II than the magnitude of ST-segment elevation in lead V2, and ST-segment depression in lead V5, suggested low probability of an LAD lesion. These findings were proposed as identifiable distinctive criteria for STEMI due to LMT lesions, and the diagnostic accuracy was equivalent also in patients without concomitant coronary diseases.

Since ECG findings in acute coronary syndromes due to LMT lesions exhibit a number of variations, it is difficult to demonstrate clear characteristics for such findings [8]. The present study population was focused on STEMI to obtain a meaningful result. Moreover, this study demonstrated that approximately 50% of such patients exhibited either RBBB or LBBB, which reflects a broad ischemic area corresponding to the broad dominant area of the myocardium supplied by the LMT. It is well known that such types of block make it difficult to identify the culprit artery in myocardial infarction by ECG. In addition, it is impossible to assess quantitative ST-segment levels, since both types of block prevent definition of the site of the J-point. Some reports on electrocardiographic features in myocardial infarction due to LMT lesions have included patients with bundle branch block [9]. We believe that such patients must be analyzed separately. The identification of ECG criteria for distinguishing LMT lesions suggested by the present study was made possible by the above two points regarding the target population, focusing on STEMI except for the patients with bundle branch block. From this point of view, our new criteria can be regarded as a novel tool that can be used to make rapid determination of LMT lesions by ECG alone.

Hirano et al. suggested that ECGs in LMT infarction can be divided into two patterns: RBBB with left axis deviation or northwest axis, and anteroseptal and lateral infarction appearance (ST-segment elevation in leads V2–5, I, and aVL) [10]. However, the present study demonstrated a wider variety of ECG patterns than in their previously proposed two patterns. This difference may be due to differences in the study population that may have included non-STEMI patients.

The present study demonstrated significant differences in ST-segment deviation between the LMT and LAD groups: (1) ST-segment elevation in lead aVR, (2) magnitude of ST-segment elevation in precordial leads, (3) magnitude of ST-segment depression in inferior leads, and (4) ST-segment depression in leads V5–6. The presence or absence of ischemia in the left circumflex artery is thought to be the cause of these differences. Generally, electrocardiographic features in STEMI due to left circumflex artery lesions are characteristically observed as ST-segment elevation in leads I, aVL, and V5–6, and ST-segment depression in leads V2–5 [11–16]. However, ST-segment deviation due to an LMT lesion does not consist of simple additions of ST-segment deviation of LAD and that of left circumflex artery; ischemia in the left and right ventricular outflow tract or the basal septum, among other factors, may influence the ECG finding.

ST-segment elevation in lead aVR is the best known ECG finding for assessment of the LMT lesion [17–20]. Yamaji et al. reported that ST-segment elevation in lead aVR was present in 88% of LMT lesions, but only 43% of LAD lesions [21]. Hence it has been considered to have high sensitivity but low specificity. Although the proportion of ST-segment elevation in lead aVR in the present study cohort was lower in all three groups (64.7% in LMT group, 11.1% in pLAD group, and 4.4% in dLAD group) than that in this prior report, the diagnostic accuracy appeared to be adequate. On the other hand, the diagnostic accuracy of our novel suggested ECG criteria, namely larger magnitude of ST-segment depression in lead II than that of ST-segment elevation in lead V2 and ST-segment depression in lead V5, can be regarded as equivalent to, or more than that in lead aVR; these criteria are particularly excellent in negative predictive value.

The present study suggested that ST-segment depression in lead V5–6 was a distinctive finding in the LMT group. It is a finding associated with ischemia in the apical area. Apical ischemia is determined by several anatomical factors: length of LAD and supply from other arteries such as large obtuse marginal branch or diagonal branch [22]. On the other hand, the magnitude of ST-segment depression in the inferior lead exhibited a significant difference across the three groups, which became gradually smaller in the order LMT > pLAD > dLAD. Although this occurs as a reciprocal change of ST-segment elevation in precordial leads, the above anatomical factors have been reported to be associated with generation of the reciprocal change. Our data agree with prior reports that reciprocal change is rare in distal LAD lesions compared to proximal LAD lesions [23–25].

The present study demonstrated other differences in findings across the three groups. The magnitude of ST-segment elevation in leads I and aVL has often been reported to exhibit differences in proximal and distal LAD lesions, and such differences are used for identifying these lesions [24]. The present study showed its frequency to be in the order of LMT > pLAD > dLAD (I, 47.1% in LMT, 26.1% in pLAD, 14.5% in dLAD; aVL, 58.8% in LMT, 40.6% in pLAD, 16.4% in dLAD). These findings may be useful in distinguishing between LMT, proximal, and distal LAD lesions.

This study has several limitations. Although the initial ECG was investigated at onset, the time from onset to recording of the ECG varied among patients. There was no comparison of a preceding ECG immediately before STEMI onset included in the analysis, since STEMI is not a scheduled event. Several baseline characteristics differed significantly between the LMT and LAD groups and this may possibly have had an impact on the ECG findings. In addition, the sample size was small.

## Conclusions

A larger magnitude of ST-segment depression in lead II than that of ST-segment elevation in lead V2 and ST-segment depression in lead V5 are proposed as identifiable distinctive ECG criteria of STEMI due to LMT lesion.

## Abbreviations

ECG: Electrocardiogram
LAD: Left anterior descending artery
LAHB: Left anterior hemiblock
LBBB: Left bundle branch block
LMT: Left main trunk
NPV: Negative predictive value
PPV: Positive predictive value
QTc: Corrected QT
RBBB: Right bundle branch block
STEMI: ST-elevation myocardial infarction
